# A stochastic two-scale model for pressure-driven flow between rough surfaces

**DOI:** 10.1098/rspa.2016.0069

**Published:** 2016-06

**Authors:** Francesc Pérez-Ràfols, Roland Larsson, Staffan Lundström, Peter Wall, Andreas Almqvist

**Affiliations:** 1 Division of Machine Elements, Luleå University of Technology, Luleå, Sweden; 2 Division of Fluid and Experimental Mechanics, Luleå University of Technology, Luleå, Sweden; 3 Division of Mathematical Sciences, Luleå University of Technology, Luleå, Sweden

**Keywords:** Reynolds equation, two scales, stochastic, contact mechanics, seals

## Abstract

Seal surface topography typically consists of global-scale geometric features as well as local-scale roughness details and homogenization-based approaches are, therefore, readily applied. These provide for resolving the global scale (large domain) with a relatively coarse mesh, while resolving the local scale (small domain) in high detail. As the total flow decreases, however, the flow pattern becomes tortuous and this requires a larger local-scale domain to obtain a converged solution. Therefore, a classical homogenization-based approach might not be feasible for simulation of very small flows. In order to study small flows, a model allowing feasibly-sized local domains, for really small flow rates, is developed. Realization was made possible by coupling the two scales with a stochastic element. Results from numerical experiments, show that the present model is in better agreement with the direct deterministic one than the conventional homogenization type of model, both quantitatively in terms of flow rate and qualitatively in reflecting the flow pattern.

## 1. Introduction

Pressure-driven flow through preamble structures such as the percolation of fluids in static seals, see e.g. [1–3] and the flow through fractured porous media, in e.g. [4,5], is a problem that has been addressed in many works before. The numerical solution of this problem is, however, complicated due to its multi-scale nature. Indeed, the flow domain is usually of the order of millimetres or even larger while the level of detail necessary to resolve the narrowest, still influential, constrictions may require nanometre order resolution [6]. As large domains resolved with a high level of detail are often required, the multi-scale nature of the flow problem prevents the usage of deterministic solutions.

Among other solutions, the problem associated with the flow through the gap between two rough surfaces this problem has repeatedly been commonly addressed in the lubrication field by separating the problem into two scales, i.e. a global scale covering the full seal domain and a local scale accounting for the roughness details. This type of scale separation was used by Christensen [7], who presented a stochastic two-scale approach, Elrod [8], who applied a perturbation technique and by Patir & Cheng [9] who presented a statistical approach and derived so-called flow factors, to model the roughness effect on the fluid flow between two rough surfaces and to link the two scales together. This concept has been further developed by others (see [10–13], for instance). Of particular interest is the homogenization technique, which provides a solid mathematical foundation to this approach (e.g. [14]). An application to the mixed lubrication problem, including a study on leakage, can be found in the two-part paper by Sahlin *et al.* [15,16]. Other, more complex cases include [17], where cavitation is included and [18], where elastohydrodynamic lubrication is considered. In the afore-mentioned mixed lubrication problem, the contact mechanics model has not been treated as a two-scale approach, instead, a periodic roughness description has been adopted [16]. This is a common approach. An exception to that is [19], where the elastohydrodynamic lubrication problem is formulated by means of separation into two scales. Two-scale approaches have turned out to be applicable in many applications, however, as the gap becomes thinner the flow becomes smaller and the local-scale model requires larger and larger domains to produce a converged value for the flow factors. This makes the two-scale approach lose its effectiveness.

The percolation problem has also been a common issue between those studying the flow through porous media. This problem is essentially equal to the one describing the pressure-driven flow through the gap between two stationary rough surfaces. In this field, it is not uncommon to find stochastic representations of permeability or the porous media itself (see, e.g. [20–24]). Generally, however, the probability distribution of the permeability is an input to the model and is not computed from actual pore-scale measurements. A model for the flow through a porous media that is similar to the model presented here, can be found in [25]. In that work the flow through a fabric pattern is investigated and the permeability distribution is generated stochastically by considering the results of computational fluid dynamics analysis over different fabric configurations.

The stochastic approach applied in porous media flow modelling is also seen as beneficial in the study of small flows between rough surfaces. A reason for this is given in the work by Dapp & Müser [26], where it is shown that the local-scale pressure drop, at very low flow rates, occurs over one very small constriction only. This implies that the local flow will depend on the geometry of such constrictions and that the total flow can only be described in a statistical manner because of the resolution requirement.

The idea behind this work is to describe the stochastic element by means of a two-scale formulation similar to those presented previously. This is done using the framework of heterogeneous multiscale method (HMM) [27]. The main novelty of this work is that it permits the estimation of the uncertainty of the results due to the random nature of the topography. Another advantage with the present model is that it is possible to restrict the size of the local domain and still obtain a converged solution. Moreover, it is shown that this approach predicts a more realistic flow pattern compared with the flow patterns obtained by using conventional two-scale models for similar local domain size.

## 2. Method

The problem to be solved is the one that governs the flow through two surfaces which are compressed against each other. As said previously, the multi-scale nature of the problem prevents the usage of a deterministic solution to such problem. Therefore, a two-scale approach is used.

In order to compute the flow rate and the flow pattern, it is first necessary to compute the deformed shape of the gap between the two surfaces (contact mechanics problem). The deformed gap is due to fluid pressure and asperity contact. In this case, we consider the fluid pressure contribution to be small and it is therefore neglected. This permits separating the problem into two smaller problems that can be solved sequentially: compute the deformed gap and compute the flow rate through that gap.

In the following, the method used in this work is presented. First, an introduction to the HMM framework, used to develop the model, is given. After that the two-scale stochastic model is introduced. This includes the theory behind the two-scale formulation of the flow model and the contact mechanics problem and the introduction of the stochastic element. Also, an overview of the solution procedure is given. Finally, the two-scale flow formulation presented in this work is compared against the well-established homogenization technique.

### (a) Heterogeneous multiscale method

The HMM is a general framework used to build two-scale models [27] which is flexible in the sense that global and local-scale models do not need to be of the same nature. This will allow us to introduce the stochastic element in a more natural way than the more rigid multi-scale homogenization technique, this is, without the imposition of periodicity in roughness. Under the HMM framework, the model is constructed into steps: (i) definition of a global-scale model, which will include some parameter or variable containing the information of the local-scale model and (ii) definition of a local-scale model that permits finding those variables or parameters. The first step is crucial as a wrong definition of the global-scale method can lead to a wrong specification of the local-scale constraints. Although it can be derived from the local-scale data, it is better to use previous (experimental or theoretical) knowledge about the global-scale behaviour. The local-scale model is usually easier to identify, as one can use a more fundamental representation of the reality. However, one must be careful on the selection of boundary conditions for the local-scale problem and the data processing used to transfer information to the global scale. In particular, the boundary conditions in the local-scale model must be specified so that the solution is consistent with the global-scale model. One should understand by consistent that the local scale should be constructed so that the global scale can be seen as a coarse representation of it.

### (b) The two-scale stochastic model

In this section, the two-scale stochastic model developed is presented. We start by introducing the two-scale methods for both the flow model and the contact mechanics problem. After this, the stochastic element is introduced in the two-scale formulation. Finally, the solution procedure is outlined.

#### (i) The two-scale flow model

Before presenting the two-scale formulation, we introduce the deterministic flow model. In this work, we consider the rectangular domain

2.1 $$\Omega_{s}:=\{x|0<x_{1}<L_{1},0<x_{2}<L_{2}\},$$

covering the full seal area. In this domain, the gap between the two rough surfaces, *h*_*ϵ*_(*x*), is resolved at a high level of detail. The subscript *ϵ* indicates that the gap oscillates rapidly due to the roughness, thus imposing a high resolution in the representation of the domain. In order to solve the fluid pressure distribution through the gap, the lubrication approximation is used. Therefore, the well-known Reynolds equation can be used. For an iso-viscous incompressible fluid and with no relative motion between the surfaces, this equation can be written as

2.2 $$\nabla \cdot (h_{\epsilon }{\left(x\right)}^{3}\nabla p_{\epsilon }(x))=0,$$

where *p*_*ϵ*_ is the fluid pressure, which will oscillate rapidly in accordance with *h*_*ϵ*_. The problem is completed by posing the boundary conditions. In order to obtain a pressure-driven flow, a pressure drop, Δ*p*, is enforced in the *x*_1_-direction by setting Dirichlet boundary conditions. In the *x*_2_-direction, periodic boundary conditions are imposed. Once the fluid pressure distribution is known, the total leak rate can be computed by integrating the mass flow over the domain *Ω*_*s*_. In accordance with the Reynolds equation, this is,

2.3 $$Q_{d}=\frac{1}{12\eta }\frac{1}{L_{1}}\int_{\Omega_{s}}h_{\epsilon }{\left(x\right)}^{3}\frac{\partial p_{\epsilon }(x)}{\partial x_{1}}\;dx$$

were *η* is the viscosity of the fluid.

As stated previously, it often becomes too computationally expensive to resolve the roughness at the global scale. Provided that the period of the oscillations is sufficiently small, one can separate the problem into two scales: one considering the detail of the oscillations (local scale) and the other accounting for the slow variations in the whole domain (global scale). Following the HMM framework, we start by presenting the global-scale model. Then we will present the local-scale one.

The global scale is solved in a domain *Ω*_*c*_, which is a coarse representation of *Ω*_*s*_. As the Reynolds equation is a mass-conservation law, it is natural to define the global-scale model also as a mass conservation law. Following a finite volume formulation, this can be posed as

2.4 $$-j_{i-1/2,j}^{0}+j_{i+1/2,j}^{0}-j_{i,j-1/2}^{0}+j_{i,j+1/2}^{0}=0,$$

where *j*^0^ are the global-scale fluxes and (*i*,*j*) identify any point of the global scale. For each grid point, the fluxes must, therefore, be estimated from the solutions of the local-scale model. We will show that, in agreement with Darcy’s law, the flux is proportional to the local pressure drop, *δp*, and we will refer to these constants as permeability, *K*. Therefore, (2.4) can be rewritten as

2.5 $$-K_{i-1/2,j}^{1}\delta p_{i-1/2,j}^{1}+K_{i+1/2,j}^{1}\delta p_{i+1/2,j}^{1}-K_{i,j-1/2}^{2}\delta p_{i,j-1/2}^{2}+K_{i,j+1/2}^{2}\delta p_{i,j+1/2}^{2}=0,$$

where

$$\begin{array}{rl} K_{i+1/2,j}^{1} & ={\left[K_{i+1/2,j}^{11}\;K_{i+1/2,j}^{12}\right]}^{T},\\ K_{i,j+1/2}^{2} & ={\left[K_{i,j+1/2}^{21}\;K_{i,j+1/2}^{22}\right]}^{T},\\ \delta p_{i+1/2,j}^{1} & =[\delta p_{i+1/2,j}^{11}\;\delta p_{i+1/2,j}^{12}]\\ \;\text{and}\;\delta p_{i,j+1/2}^{2} & =[\delta p_{i,j+1/2}^{21}\;\delta p_{i,j+1/2}^{22}]. \end{array}$$

Here the first superscript indicates the direction of the corresponding flux and the second one the direction of the local pressure drop. Note that, in order to express the flux in m^3^ s^−1^ and the permeability in m^3^, (2.5) is scaled by the (constant) viscosity. Together with the boundary conditions, posed equally to those in the deterministic problem, (2.5) permits computing the global-scale fluid pressure. The total leak rate is then

2.6 $$Q_{2s}=\frac{1}{\eta }\sum_{\partial^{o}\Omega_{c}}K_{i+1/2,j}^{11}\delta p_{i+1/2,j}^{11},$$

where ∂^*o*^*Ω*_*c*_ is the part of the boundary of *Ω*_*c*_ regarded as an outlet.

In the global-scale model, the permeabilities are the parameters containing the local-scale information. We, therefore, define the local scale in order to compute them. Let us start by deriving the fluxes in (2.4). As an example, consider $j_{i+1/2,j}^{0}$; the others can be defined analogously. In order to do so, we define a local domain between the neighbour global-scale points,

2.7 $$\omega =\{x|X_{1\;i,j}<x_{1}<X_{1\;i+1,j},X_{2\;i,j-1/2}<x_{2}<X_{2\;i,j+1/2}\},$$

which has a size Δ*x*_1_×Δ*x*_2_. Similarly as in the deterministic formulation, we solve the fluid pressure distribution by means of the Reynolds equation (2.2). We, however, need to pose the boundary conditions in a way that the local-scale model is consistent with the global-scale one. In order to see which conditions must be fulfilled, we assume that the global-scale component of *p*_*ϵ*_, *p*^0^, varies linearly between the global-scale points, i.e.

2.8 $$p^{0}(x)=P_{i,j}^{0}+\frac{P_{i+1,j}^{0}-P_{i,j}^{0}}{\Delta x_{1}}(x_{1}-X_{1\;i,j})+\frac{P_{i+1/2,j-1/2}^{0}-P_{i+1/2,j+1/2}^{0}}{\Delta x_{2}}(x_{2}-X_{2\;i,j}),$$

where the capital letters refer to the discrete representation in the global scale. In order for it to be consistent, the local-scale solution should be in agreement with the above representation. Therefore, it should satisfy

2.9 $$\left\langle \frac{\partial p_{\epsilon }}{\partial x_{1}}\right\rangle =\frac{\delta p_{i+1/2,j}^{11}}{\Delta x_{1}},\;\left\langle \frac{\partial p_{\epsilon }}{\partial x_{2}}\right\rangle =\frac{\delta p_{i+1/2,j}^{12}}{\Delta x_{2}},$$

where

$$\begin{array}{rl} \delta p_{i+1/2,j}^{11} & =P_{i+1,j}^{0}-P_{i,j}^{0},\\ \delta p_{i+1/2,j}^{12} & =P_{i+1/2,j+1/2}^{0}-P_{i+1/2,j-1/2}^{0} \end{array}$$

and 〈⋅〉 indicates average over the local domain. Other conditions over the mean value of *p*_*ϵ*_ could be introduced, but the flux would not be affected. Several boundary conditions can be posed so that the constraint (2.9) is satisfied. Among them, the one providing better results is the near periodic condition, i.e. periodicity of *p*_*ϵ*_−*p*^0^ [28].

The boundary condition selected, however, introduces an unwanted coupling between the global and the local scale. In order to remove this coupling, we start by defining $p_{\epsilon }=p_{\epsilon }^{1}+p_{\epsilon }^{2}$, with $p_{\epsilon }^{1}$, near periodic in the *x*_1_-direction and periodic in the *x*_2_-direction, and $p_{\epsilon }^{2}$, defined analogously. Furthermore, we define the non-dimensional variables

$$\begin{array}{rl} y^{1} & =\frac{\left(x-X_{i,j}\right)}{\Delta x_{1}},\\ y^{2} & =\frac{\left(x-X_{i,j}\right)}{\Delta x_{2}},\\ p^{1} & =\frac{p_{\epsilon }^{1}}{\delta p_{i+1/2,j}^{11}}\\ \;\text{and}\;p^{2} & =\frac{p_{\epsilon }^{2}}{\delta p_{i+1/2,j}^{12}}. \end{array}$$

Therefore, (2.2) can be written in *ω* as

2.10 $$\frac{\delta p_{i+1/2,j}^{11}}{\Delta x_{1}}\nabla \cdot (h_{\epsilon }{\left(y^{1}\right)}^{3}\nabla p^{1}(y^{1}))+\frac{\delta p_{i+1/2,j}^{12}}{2\Delta x_{2}}\nabla \cdot (h_{\epsilon }{\left(y^{2}\right)}^{3}\nabla p^{2}(y^{2}))=0.$$

We can now find a solution by setting the two terms equal to zero independently and thus obtain two problems, both of which are independent from the global scale, i.e.

2.11*a* $$\nabla \cdot (h_{\epsilon }{\left(y^{1}\right)}^{3}\nabla p^{1}(y^{1}))=0,\;p^{1}(y)-y_{1}^{1}\;\text{periodic},$$

and

2.11*b* $$\nabla \cdot (h_{\epsilon }{\left(y^{2}\right)}^{3}\nabla p^{2}(y^{2}))=0,\;p^{2}(y)-y_{2}^{2}\;\text{periodic}.$$

It can be seen that the solution obtained by combining this two problems satisfies the condition (2.9). Once the local problems are solved, we can compute the local flux, i.e.

2.12 $$\begin{array}{rl} j_{i+1/2,j}^{0} & =\frac{1}{12\eta \Delta x_{1}}\int_{\omega }h_{\epsilon }{\left(x\right)}^{3}\frac{\partial p_{\epsilon }(x)}{\partial x_{1}}\;dx\\ & =\frac{\delta p_{i+1/2,j}^{11}}{12\eta }\int_{\omega }h_{\epsilon }{\left(y^{1}\right)}^{3}\frac{\partial p^{1}(y^{1})}{\partial y_{1}^{1}}\;dy^{1}+\frac{\delta p_{i+1/2,j}^{12}}{12\eta }\frac{\Delta x_{2}}{\Delta x_{1}}\int_{\omega }h_{\epsilon }{\left(y^{2}\right)}^{3}\frac{\partial p^{2}(y^{2})}{\partial y_{1}^{2}}\;dy^{2}. \end{array}$$

We can now identify the local- and the global-scale contribution to the flux. By defining the permeability, *K* as the local-scale contribution, we can write

2.13 $$\frac{j_{i+1/2,j}^{0}}{\eta }=K_{i+1/2,j}^{11}\delta p_{i+1/2,j}^{1}+K_{i+1/2,j}^{12}\delta p_{i+1/2,j}^{2},$$

which leads to the global-scale model (2.5).

#### (ii) The two-scale contact mechanics model

The fluid flows through the gaps, *h*_*ϵ*_, between the contacting surfaces. This gap depends on the applied load. The flow defined previously is solved on a clearance between the surfaces, *h*_*ϵ*_, which varies depending on the applied load. Therefore, the deformed shape of the gap must be computed before the flow problem can be assessed. In order to do so, a model based on the one presented in [15], is used. The equations governing this method can be posed as

2.14*a* $$\begin{array}{rl} h_{\epsilon }(x) & =h_{1}(x)+u(x)+g_{00}, \end{array}$$

2.14*b* $$\begin{array}{rl} u(x) & =\frac{1}{E^{*}}\int_{\Omega_{s}}\frac{p_{c}(x)}{\sqrt{{\left(x_{1}-x_{1}^{\prime }\right)}^{2}+{\left(x_{2}-x_{2}^{\prime }\right)}^{2}}}\;dx^{\prime }=\int_{\Omega_{s}}k(x-x^{\prime })p_{c}(x)\;dx^{\prime }, \end{array}$$

2.14*c* $$\begin{array}{rl} p_{c}(x)\cdot h_{\epsilon }(x) & =0,\;0\leq p_{c}(x)\leq H,\;0\leq h_{\epsilon }(x) \end{array}$$

2.14*d* $$\begin{array}{rl} \;\text{and}\;W & =\frac{1}{A_{\Omega_{s}}}\iint_{\Omega_{s}}p_{c}(x)\;dx, \end{array}$$

where the clearance, *h*_*ϵ*_, is defined as the original (no pressure) gap, *h*_0_, plus the deformation, *u*, caused due to the contact pressure, *p*_c_, and a rigid body movement, *g*_00_. The deformation, *u*, is computed by the convolution of a coefficients kernel, *k*, and the contact pressure, *p*_c_. This can be seen as adding the deformation caused by the pressure at all points. The equivalent Young modulus, E*, is defined as

2.15 $$E^{*}={\left(\frac{1-\nu_{1}^{2}}{E_{1}}+\frac{1-\nu_{2}^{2}}{E_{2}}\right)}^{-1},$$

where *E*_*i*_ and *ν*_*i*_ are the Young modulus and Poisson ratio of each surface. Equation (2.14c) establishes a complementarity relation between *h*_*ϵ*_ and *p*_c_, meaning that when there is contact, the clearance is zero and when there is no contact, the pressure is zero. Finally (2.14d) imposes that the average contact pressure is equal to a given total load, *W*. A perfect plasticity condition is defined by imposing *p*_c_≤*H*, where *H* is the hardness of the softer material. This problem can be solved by using the algorithm presented in [15], modified to account for the non-periodicity in the *x*_1_-direction (e.g. [29]).

In the same way as in the fluid computation, the computational time for the contact mechanics problem rises as the domain size grows. Therefore, a two-scale solution is also desired. In order to justify the two-scale formulation of this problem, we need to justify that (i) it is a correct approximation to use a coarser representation of the surface to capture the global-scale trends in the contact mechanics results and (ii) that it is a correct approximation to compute the contact mechanics problem in the local cells by using the nominal pressure *p*_nom_=*W*_local_ as the only information from the global scale.

We start by justifying the point (i). The goal is to show that the utilization of a coarse representation of *h*_0_ gives as a result a coarse representation of *h*_*ϵ*_ and *p*_c_. To do so, we apply a Gaussian filter, *g*, to (2.14a)–(2.14d). We define

2.16 $$\hat{f}(\xi )=\int f(x)\;e^{-2\pi ix\xi }\;dx,$$

as the Fourier transform of a function *f*(*x*). The equations then read

2.17*a* $$\begin{array}{rl} \hat{H}(\xi ) & =\hat{g}(\xi )\cdot {\hat{h}}_{\epsilon }(\xi )=\hat{g}(\xi )\cdot ({\hat{h}}_{0}(\xi )+\hat{u}(\xi )+{\hat{g}}_{00}(\xi ))={\hat{H}}_{0}(\xi )+\hat{U}(\xi )+{\hat{G}}_{00}(\xi ), \end{array}$$

2.17*b* $$\begin{array}{rl} \hat{U}(\xi ) & =\hat{g}(\xi )\cdot \hat{u}(\xi )=\hat{k}(\xi )\cdot \hat{g}(\xi )\cdot {\hat{p}}_{c}(\xi )=\hat{k}(\xi )\cdot {\hat{P}}_{c}(\xi ) \end{array}$$

2.17*c* $$\begin{array}{rl} \;\text{and}\;W & =\frac{1}{A_{n}}\iint_{\Omega_{s}}\hat{G}(\xi )\cdot {\hat{p}}_{c}(\xi )\;dx=\frac{1}{A_{n}}\iint_{\Omega_{s}}{\hat{P}}_{c}(\xi )(x)\;dx, \end{array}$$

where the capital letters identify the filtered functions. In order to obtain (2.17c), the property of the filter to maintain the mean value has been used. Equation (2.14c) has not been added because it does not hold exactly for the coarsened problem. If one think on the complementarity condition and how the filter works, one can see that it holds approximately provided that the coarsening does not alter the geometry at the global scale. Regarding the plastic limit, the pressure is expected to be lower, and in a more spread area due to the filtering. Therefore, there should be less plastic deformation. This, however, should not significantly affect the result.

Unlike the flow case, where each local cell corresponds to one point in the global scale, this is not the case in this coarse representation. In order to obtain the nominal pressure, *p*_nom_, of a local cell, the global-scale result should be averaged over a region of equivalent size.

We now discuss about the correctness of the second statement (ii). It has been shown that the elastic deformation caused by pressure is a long-range effect and it cannot be neglected [30]. However, the requirement here is smaller. The deformation on the local domain needs to be computed only up to a constant (*g*_00_). Therefore, by assuming that the elastic deformation caused by pressures far-away from the local domain has a constant value over the considered domain, the local scale contact mechanics problem can be solved independently from the global domain.

In order to assess the validity of this assumption one should note that, according to the St. Vennant’s principle, the contribution of the distant regions is smooth. Then, by assuming that the local cell is reasonably away from the boundaries, similar deformation can be assumed in all directions, leading to a flat overall deformation. Although crude, it is an adequate first approximation. The assumption of flat deformation is clearly not adequate if one refers to the deformation on points near the cell boundaries caused by pressures in points neighbouring the local cell. In such close points, however, it is a reasonable claim that the roughness is similar to that on the cell. Therefore, a periodic roughness assumption is also reasonable.

#### (iii) The stochastic approach

In the previous sections, a two-scale model has been developed. The stochastic element has not, however, been introduced yet. Indeed, following the presented procedure, four cell problems (two for each direction) must be solved for each grid node in the global scale. Moreover, the results can be sensitive to the measurement used for the computations. Because the surface topography is a random process, two different measurements of the same surface (or two equivalent ones) are expected to give different results. It is, therefore, of greater interest to obtain the results in the form of an expected value plus a confidence interval rather than simply a given value.

If one now takes a look at the permeabilities computed one notices that they can be modelled as a random variable following a log-normal distribution. This distribution is, however, rather broad. Moreover, it is expected to see some spatial correlation between the different permeabilities, caused by fluctuations in the global scale. Therefore, a reference parameter, either average interfacial separation, $\bar{h}$, or nominal pressure, *p*_nom_, is taken from the global-scale contact mechanics computation and the permeabilities are fitted to different log-normal distributions as a function of these parameters. These distributions have a narrower permeability range and, therefore, are more meaningful.

Following this approach, the global scale is therefore not constructed by solving the local problem on each coarse grid point but by randomly generating a permeability value for each of those points. In order to ensure sufficient accuracy, numerous realizations of the global scale are computed following a Monte Carlo approach. The output is, therefore, given as a probability distribution instead of a single value.

It is worth noticing that modelling a gap between two rough surfaces by randomly assign permeabilities from a log-normal distribution (or other similar distributions) is a common practice in the porous media literature (e.g. [20,31]). With the present approach, the parameters for such distribution are no longer obtained experimentally but from modelling the problem at the local scale.

#### (iv) Solution procedure

**Figure 1.**
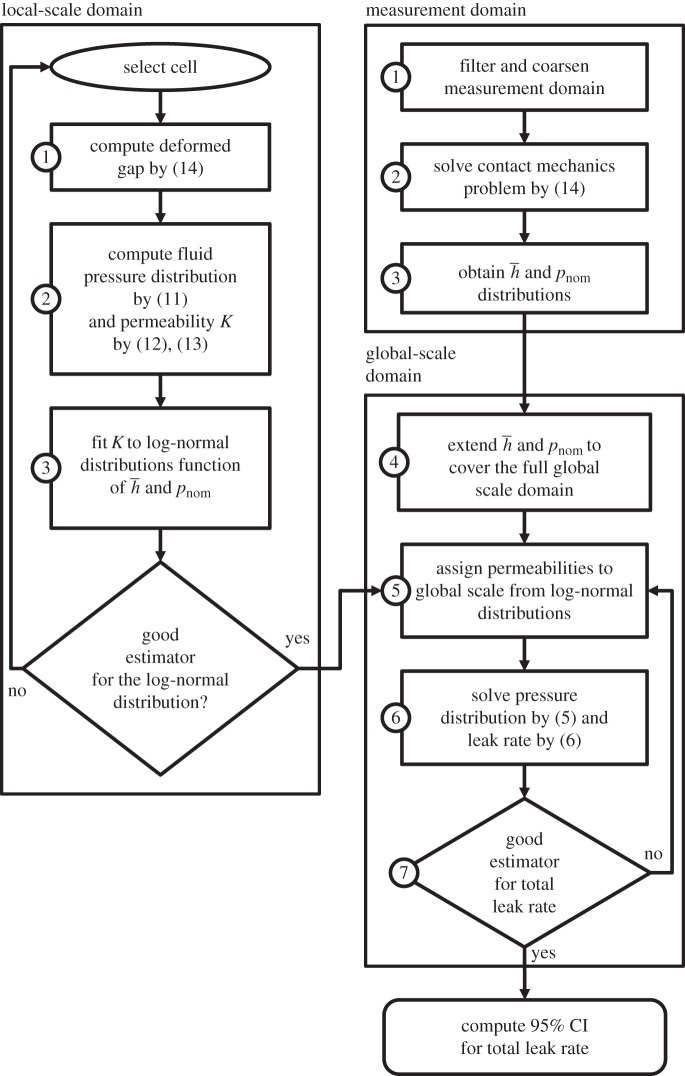
Flow chart of the proposed algorithm, indicating the steps and the domains to be used. CI, confidence interval.

**Figure 2.**
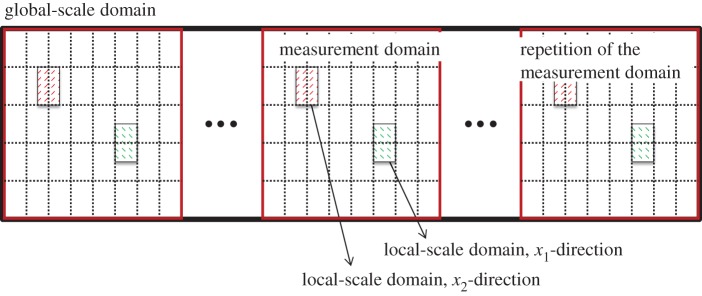
Representation of the different domains used in the implementation of the model. (Online version in colour.)

A flow chart of the solution procedure for the model is depicted in figure 1. In the following, a more detailed description is given. Both scales are effectively separated during the computations and therefore they are treated separately here also. For clarity, the different domains considered are represented in figure 2

**Figure 3.**
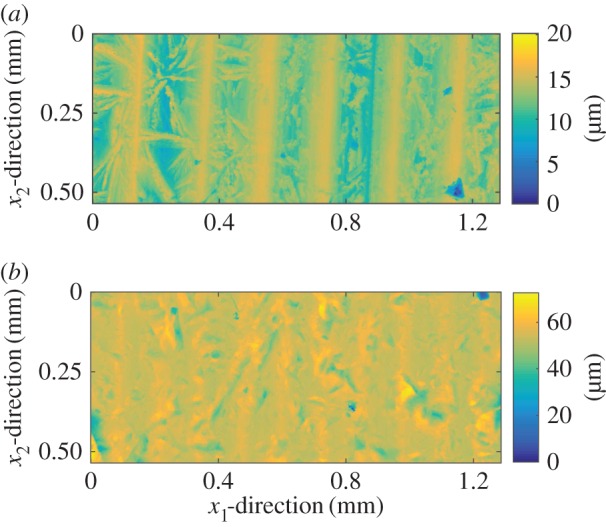
The topographies used in the studies performed in this work: (*a*) Topography 1, corresponding to a turned surface and (*b*) Topography 2, corresponding to a sand-blasted surface. (Online version in colour.)

We start by considering the local-scale solution procedure. In order to solve the local problems, the whole measured domain is divided into a set of local cells of size Δ*x*_1_×Δ*x*_2_. For the following numerical analysis, these cells are mirrored in order to obtain a periodic roughness. We note that this implies that the permeabilities *K*^12^ and *K*^21^ will be zero (see [32] for a justification). However, even without mirroring, these are expected to be small in most cases, also in the surface textures used in this work (figure 3; this should be reconsidered the model is applied to surfaces with different textures). The solution procedure is then as follows:

(i) The deformed gap in the local domain is computed for a range of nominal pressures *p*_nom_, as described in §2b(ii). It is advised to compute the deformation for a very small nominal pressure, which shall serve as a zero value during the interpolation.
(ii) The fluid pressure distribution is computed by (2.11) and the permeability is computed according to (2.12) and (2.13). This is done for the range of nominal pressures previously computed and for a specified range of average interfacial separations $\bar{h}$. A combination of Dirichlet boundary condition in the direction of the pressure drop and homogeneous Neumann boundary conditions in the transverse direction is used instead of the near periodic condition. The reason for this is that this combination is easier to implement and yet equivalent (see [32] for details).
(iii) The distribution of permeabilities for the given set of cells, and for each reference parameter (*p*_nom_ or $\bar{h}$) are fitted to a log-normal distribution. The set of cells should be large enough in order to obtain a good estimate for the parameters of the log-normal distribution. A random selection of cells is desired to increase robustness against any possible spacial variation of permeability.

Once the permeabilities are obtained from the local-scale, the global-scale model is implemented in the following steps:

(i) A measurement of the topography is used as the input. This measurement domain, *Ω*, is filtered by using a Gaussian filter and re-sampled to a coarser grid.
(ii) The contact mechanics problem is solved using the coarse representation of the measurement as input.
(iii) The resulting pressure and interfacial separation distribution is further coarsened by averaging over domains of the same size of the local-scale cells. This results in a nominal pressure and an average interfacial separation distribution. It is important to select the same size as of the local cells as the permeability distributions might depend on the size.
(iv) The global domain is covered by repetition of the nominal pressure and average interfacial separation distributions. A global-scale variation might be included in the form of a nominal pressure or average interfacial separation global-scale variation. In order to achieve this, the extended domain is obtained for a range of global nominal pressure or average interfacial separation. The global scale is then obtained by interpolating these extended distributions in accordance with the global-scale trend.
(v) Permeabilities are assigned to each point in the global scale. To do so, the parameters of a log-normal distribution are first assigned to each point by interpolation based on the reference parameter (*p*_nom_ or $\bar{h}$). Then a permeability value is generated using that distribution.
(vi) The global scale fluid pressure distribution is computed by means of (2.5) and the total leak rate is computed by means of (2.6).
(vii) Points 5 and 6 are repeated until good estimates for the leak rate and its variance are achieved. If the leak rate does not follow a normal distribution, the 95% CI can be computed by performing a large-enough number of realizations so that 95% of them have a leak rate which consistently falls inside a fixed range.

It is important to note that there will be cells with zero local permeability, occurring when there is no available path connecting the inlet and the outlet in the local domain. Those cells cannot be included in the log-normal distribution. Instead, the fraction of cells with zero permeability is stored. Then a number of cells corresponding to this fraction is set to zero in the global scale.

The reference parameter should be chosen between the nominal pressure and the average interfacial separation as the one that better correlates with the permeability. For example, for the turned surface with the topographies depicted in figure 3, permeabilities in the *x*_1_-direction correlate well with nominal pressure (except for when contact is lost) while the one in the *x*_2_-direction correlate better with the average interfacial separation.

### (c) An average permeability approach

Most of the most successful two-scale models for fluid flow are based on the homogenization technique. Therefore, we benchmark our flow model to it. We first note that the proposed boundary conditions make the problem equivalent to that of standard homogenization. Indeed, by making the change of variables $\chi^{1}=p^{1}+y_{1}^{1}$ and $\chi^{2}=p^{2}+y_{2}^{2}$, the local problems read

2.18*a* $$\nabla \cdot (h_{\epsilon }^{3}\nabla \chi^{1})=\frac{\partial h_{\epsilon }^{3}}{y_{1}^{1}},\;\chi^{1}\;\text{periodic}$$

and

2.18*b* $$\nabla \cdot (h_{\epsilon }^{3}\nabla \chi^{2})=\frac{\partial h_{\epsilon }^{3}}{y_{1}^{2}},\;\chi^{2}\;\text{periodic}$$

and the flux reads

2.19 $$j_{i+1/2,j}^{0}=\delta p_{i+1/2,j}^{11}\frac{1}{12\Delta x_{1}}\int_{\omega }h_{\epsilon }^{3}\left(\frac{\partial \chi^{1}}{\partial y_{1}^{1}}-1\right)dy^{1}+\delta p_{i+1/2,j}^{12}\frac{1}{12\Delta x_{2}}\int_{\omega }h_{\epsilon }^{3}\frac{\partial \chi^{2}}{\partial y_{1}^{2}}\;dy^{2},$$

which is equivalent to the homogenized results by reinterpreting the flow factors *a*_11_ and *a*_21_ as the local permeabilities *K*^11^ and *K*^21^ (e.g. [10]). We note that this resemblance was already studied in [32] and that a more rigorous derivation of the consistency between the HMM formulation and the standard homogenization is given in [33] for the more general time-dependent case.

The main difference lies then in the assumptions regarding the local-scale roughness. In homogenization it is assumed to be periodic, therefore, it is natural to assume that the flow factors (or permeability) are constant or vary smoothly in the global scale or, at least, in sub-domains of the global scale much larger than the global-scale size. One then obtains the values by averaging several cell realizations. Hereafter, we will refer to this procedure as the averaged permeability approach. By contrast, the periodic boundaries in the given approach represent only a consistent way to couple the two scales. This allows accounting naturally for spacial variation of permeability. We have used this feature to introduce a stochastic representation of the surface in §2b(iii).

In the average permeability approach, the pressure-driven flow problem considered will lead to a constant pressure gradient in the *x*_1_-direction and a zero pressure gradient in the *x*_2_-direction. The total leak rate, *Q*_*H*_, is therefore

2.20 $$Q_{H}=\frac{\Delta p}{\eta }{\bar{K}}_{H}\frac{L_{2}}{L_{1}},$$

where ${\bar{K}}_{H}$ is the average permeability in the *x*_1_-direction.

## 3. Results

The performance of the model is shown in two steps. First, the two-scale model is validated against a deterministic solution. Then, an example of its usage, the model is applied to a test case.

In both cases, two different topographies are used, as shown in figure 3. Topography 1 is highly anisotropic and corresponds to a turned surface. Topography 2 has been sand-blasted to obtain a more isotropic surface (although still with a clear anisotropic component). These topographies have also been chosen in order to obtain two clearly different total flow rates. The leakage studied is the one occurring between these two surfaces and a flat surface. The boundary conditions posed in the global scale (and in the deterministic solution) are periodic in the *x*_2_-direction and Dirichlet in the *x*_1_-direction, in order to impose a pressure drop Δ*p*. The topography used is mirrored in the *x*_2_-direction in order to avoid a jump in topography when applying the periodic boundaries.

The material, assumed to be linear elastic-perfectly plastic, is described by the parameters *E*_1_=*E*_2_=206 GPa, *ν*_1_=*ν*_2_=0.3 and *H*=2.75 GPa. The leak rate is computed by (2.3), (2.6) or (2.20), depending on the methodology. In the presented results, however, it is scaled by the factor *η*/Δ*p*. The Gaussian filter used for coarsening have a cut-off length 0.01 times the total length of the measurement and the filtered surface is re-sampled to one-sixteenth of the previous number of points. The original lateral resolution for the contact mechanics (both for the deterministic and the local-scale cells) is of 0.896 μm, which corresponds to a grid size of 600×1440 nodes for the topographies presented in figure 3. The tolerance for the load is set to 1×10^−3^% and the one for the contact plane is set to 10^−10^ m (see points 4 and 8 of the algorithm presented in [15] for reference).

### (a) Two-scale model validation

In order to do the validation the measured domain *Ω* is separated into a set of local cells, *ω*. The size of the local cells in the *x*_1_-direction, Δ*x*_1_ is taken so that each cell corresponds to one wavelength of the main frequency in Topography 1 (figure 3). In order to facilitate comparison, the same sizes have been also used for Topography 2. This means that the measurement domain is divided into six cells, each of which with a length of 0.21 mm. In order to verify convergence with the domain size, three widths in the *x*_2_-direction, Δ*x*_2_, are taken. These are 0.18, 0.09 and 0.045 mm respectively, giving 6, 12 and 24 local-scale cells in that direction. A typical two-scale formulation requires the local domain to be at least one order of magnitude smaller that the global domain [34], which is not fulfilled in the present validation. This is because of the restriction on the measurement domain size. We note, however, that the error obtained when using a larger domain is expected to be smaller. Therefore, the validation can be directly extended to the more common situation where the two-scale separation is clearer.

**Figure 4.**
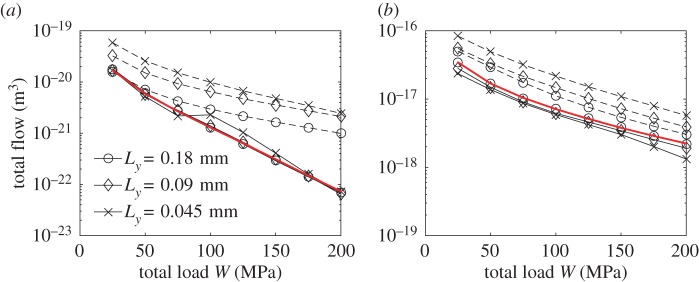
Comparison of the leak rate between the deterministic solution (red) and the two-scale approaches using (*a*) Topography 1 and (*b*) Topography 2. The results of the present model are given in solid line while theones coming from the averaged permeability approach, described in §3c, are depicted with a dashed line. (Online version in colour.)

The three techniques presented in §§2b and c are compared. In all three cases, the contact mechanics model described in §2 is used to compute the deformed gap. The deterministic approach for contact mechanics is used to compute the deterministic flow, while the two-scale contact mechanics approach is used for the other two techniques. The results for a range of total load *W* and for the three different widths Δ*x*_2_ are depicted in figure 4.

The present two-scale model gives a solution that is in good agreement with the deterministic one for both tested topographies, specially for the two wider local cells. The average permeability approach predicts generally a higher leak rate. Although it could be acceptable for Topography 2 when using the higher width, this is not true for Topography 1. Moreover, for a given cell size, the present two-scale model gives significantly better accuracy. The error between the present two-scale formulation and the deterministic solution, defined as

**Figure 5.**
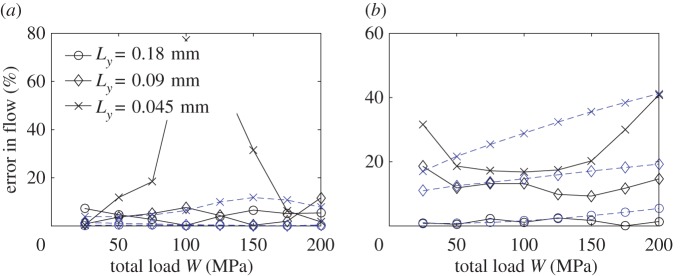
Error between the solutions of the present two-scale model and the deterministic solution, using (*a*) Topography 1 and (*b*) Topography 2 . Black solid lines show the results with the full two-scale model while the blue dashed linesshow the results computed using the deterministic solution of the contact mechanics problem. (Online version in colour.)

**Figure 6.**
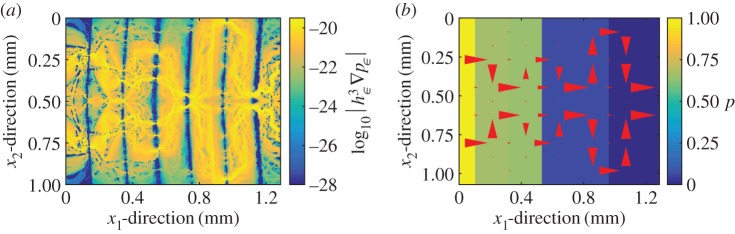
Comparison of the flow pattern for the deterministic and the present two-scale method for the pressure-driven flow (with unitary pressure drop) through the gap obtained by applying to Topography 1 a total load *W* of 200 MPa.(*a*) Absolute value of deterministic flux, in logarithmic scale. (*b*) Pressure distribution and flux computed by the present two-scales method. The size and direction of the red arrows corresponds to the flux. Cells of width 0.18 mm have been used. (Online version in colour.)

**Figure 7.**
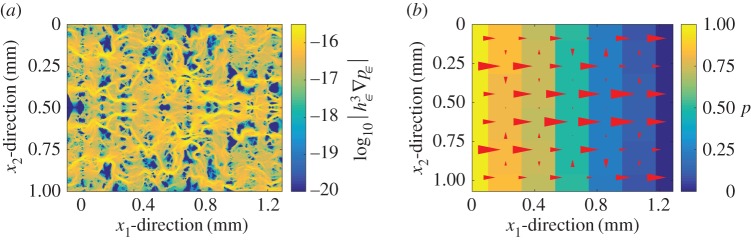
Comparison of the flow pattern for the deterministic and the present two-scale method for the pressure-driven flow (with unitary pressure drop) through the gap obtained byapplying to Topography 2 a total load *W* of 200 MPa. (*a*) Absolute value of deterministic flux, in logarithmic scale. (*b*) Pressure distribution and flux computed by the present two-scales method. The size and direction of the red arrows corresponds to the flux. Cells of width 0.18 mm have been used. (Online version in colour.)

3.1 $$\frac{|Q_{2s}-Q_{d}|}{Q_{d}},$$

is depicted in figure 5. It can be observed that reasonable values are obtained for the two wider widths (maximum error for Topography 1 using cells of width 0.18 and 0.09 mm is 7 and 11%, respectively, and 5.5 and 19% for Topography 2 and the same widths). In order to identify the main source of error, the error between the deterministic solution and the solution with the present two-scale fluid model using the deterministic computation for the gap as the flow domain is also depicted. For Topography 1, it can be seen that it is much smaller, which identifies the two-scales contact mechanics model as the main source of error for this case. For Topography 2, however, this is not the case. The reason given is that due to the cell selection made for Topography 1, the boundary conditions in the flow model are particularly well suited, as the pressure becomes nearly constant at the edges of the domain due to the larger gap. This is, however, not the case for Topography 2. Thus, it becomes clear that the error and its main source is topography dependent. Despite that, given a careful choice of domain size, a sufficiently good approximation can always be made. The average permeability approach based on the homogenization technique has been proved to produce good results for similar pressure-driven conditions [16]. The discrepancy observed in this work is, therefore, attributed to the small size of the local cells, which makes them not representative of the topography. This is further enhanced when the leak rate is reduced. In order to see why the local-scale cells are not representative when using the average permeability approach but are representative in the present two-scale formulation, one can analyse the flow pattern. The flow pattern obtained using Topography 1, in terms of deterministic flux, is depicted in figure 6*a* for a high total load *W*, i.e. for a small leak rate. It can be seen that the fluid follows a particular pattern through the gap. Whenever a path is available, it advances in the *x*_1_-direction, which is the direction of the pressure gradient. When this is not possible, or when the path is too small, the flow advances in the *x*_2_-direction until a large path is found and flow in the *x*_1_-direction is possible again. Furthermore, the advance in the *x*_2_-direction is as large as the half of the width of *Ω*, which is the maximum value due to the surface has been mirrored. In fact, one should expect even larger advances in this direction when computing over a larger domain. This implies that a representative cell for the average permeability approach would need to be at least as large as *Ω* for it to be possible to capture the flow pattern correctly. In the present two-scale formulation, however, the heterogeneity in the permeability distribution can enforce such flows in the *x*_2_-direction even when using small local cells. This can be seen in figure 6*b*, where it can be observed that the flow pattern is satisfactorily captured by the present two-scales approach. Another fundamental difference of the two approaches lies in how much large values of permeability affect the total leak rate. In the average permeability approach the cells with high permeability will increase the average permeability and therefore significantly affect the total leak rate. In the present two-scale formulation, the leak rate is controlled by the narrowest constriction that must be crossed. This is, in fact, a more correct representation of the problem [26]. Therefore, the influence of the high permeability values is not as large as in the average permeability approach. This is also the explanation for the observed differences in the leak rate prediction shown in figure 4. In figure 7, a similar comparison, using Topography 2, is depicted. The present two-scale approach captures, again the correct flow pattern. In this case, however, the flow in the *x*_2_-direction is much less important (although significant enough to affect the results) and the constrictions are more evenly spread. This, in turn, explains why the average permeability approach does not deviate as much as in the previous case.

It is important to emphasize that the average permeability approach only predicts a too high leak rate when it is small, i.e. at high total load. Otherwise, the variation in the permeabilities are not expected to be large and the flow perpendicular to the direction of the pressure drop is expected to be less important. In those cases, the average permeability approach can be considered a good approximation. This can be seen in figure 4, where the prediction from the average permeability approach is close to the deterministic one at the lowest total load and the deviation observed when using Topography 2, which exhibits a higher leakage, is smaller.

### (b) Two-scale stochastic model

Once having validated the two-scale part of the model, we show in this section the performance of the model by applying it to a case example. The measurement domain used for this is the same as in the previous section.

**Figure 8.**
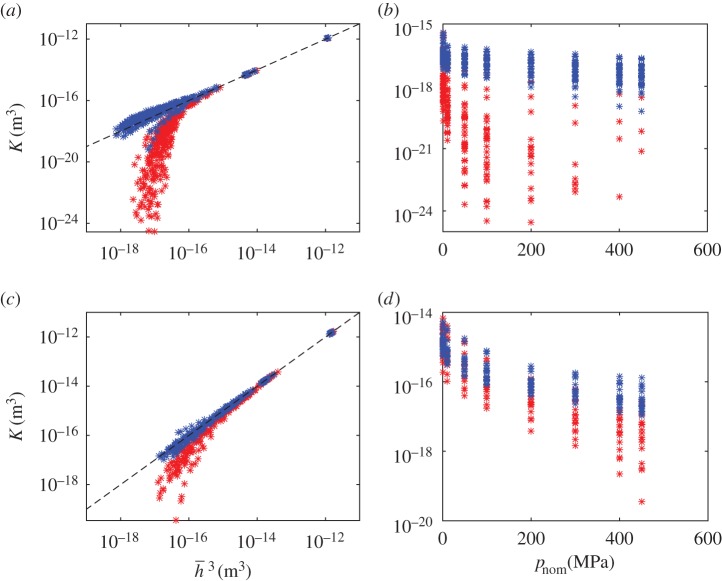
Permeability versus the two reference parameters (average interfacial separation, $\bar{h}$ and nominal pressure, *p*_nom_). (*a*,*b*) Permeabilities corresponding to Topography 1, with a cell width of 0.09 mm. (*c*,*d*) Permeabilities corresponding to Topography 2, with a cell width of 0.18 mm. Permeabilities in the *x*_1_-direction aredepicted in red and those in the *x*_2_-direction in blue. The black dashed line depicted in (*a*,*c*) corresponds to the equality $k={\bar{h}}^{3}$. The cells with zero permeability are not depicted. (Online version in colour.)

Let’s start by considering the local-scale results. Having computed the permeabilities for the set of local cells, these values must be fitted to log-normal distributions. In order to decide which reference parameter to use, we study the correlation between the permeability and the two reference parameters (nominal pressure and average interfacial separation). These relations are shown in figure 8. Focusing first on the correlation between average interfacial separation and permeability, one can observe that the permeabilities in the *x*_2_-direction can be approximated by $K\approx {\bar{h}}^{3}$. The same trend is followed by permeabilities in the *x*_1_-direction when there is no contact and the separation is large. This approximation will be used for simplicity when obtaining the permeability distributions in the global scale. When the nominal pressure increases, the permeability in the *x*_1_-direction is reduced significantly (note here that a large number of cells have zero permeability at high nominal pressures), while the average interfacial separation remains nearly constant. Therefore, we use the nominal pressure instead of average interfacial separation as the reference parameter. As seen in figure 8, there is a large spread in the correlation between permeability and nominal pressure, but one can clearly observe a trend in both mean value (decreasing) and spread (increasing) of permeability. The percentage of cells with zero permeability also increases with increasing nominal pressure. In order to describe the permeability in the *x*_1_-direction log-normal distributions are fitted for each value of the nominal pressure.

**Figure 9.**
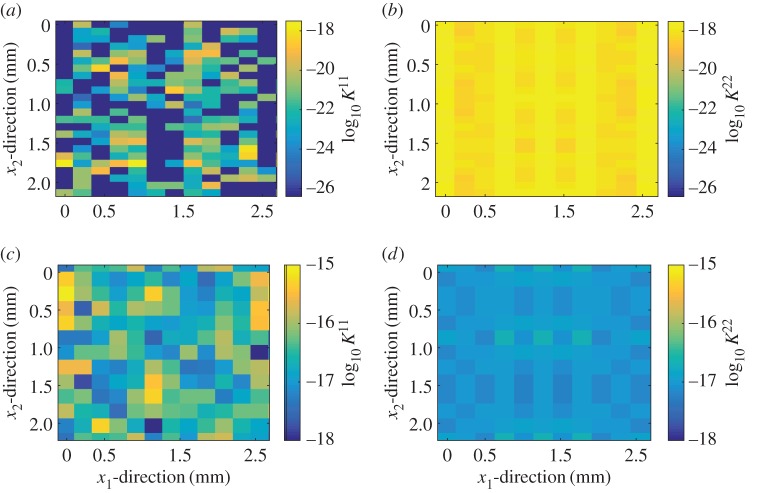
Example of one realization of the permeability distributions in both directions. (*a*,*b*) Topography 1, whereas (*c*,*d*) Topography 2. The global-scale domain has four times the size of the measurement domain (see figure 2 for measurement domain specification). (Online version in colour.)

**Figure 10.**
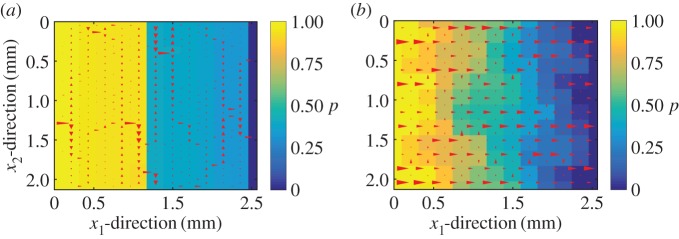
Example of a flow pattern, corresponding to the realizations depicted in figure 9. (*a*) Topography 1 and (*b*) Topography 2. The size and direction of the red arrows correspondsto the flux. (Online version in colour.)

The log-normal distributions relating permeability and nominal pressure and the approximation $K\approx {\bar{h}}^{3}$ are used to build the permeability distributions and the global scale leak rate is computed. An example of a permeability distribution is shown in figure 9 and the resulting flow pattern for a unitary pressure drop in the *x*_1_-direction is depicted in figure 10. It can be seen in figure 10*a* how the flow advances in the *x*_2_-direction until an easy path is found in the *x*_1_-direction. For the case of Topography 2 (figure 10*b*) the leakage is much higher, resulting in a less important flow in the *x*_2_-direction. One could define this flow as advancing in the *x*_1_-direction until a blockage is found, then doing a small shift in the *x*_2_-direction to avoid it and continue in the *x*_1_-direction.

One can think on these flow patterns, particularly the one corresponding to Topography 1, similarly to the description given by Persson & Yang [2], where the flow domain is described as a network of paths with critical constrictions randomly located. In the present model, however, no assumption on either the distribution or the size of the paths and the constrictions needs to be imposed.

**Figure 11.**
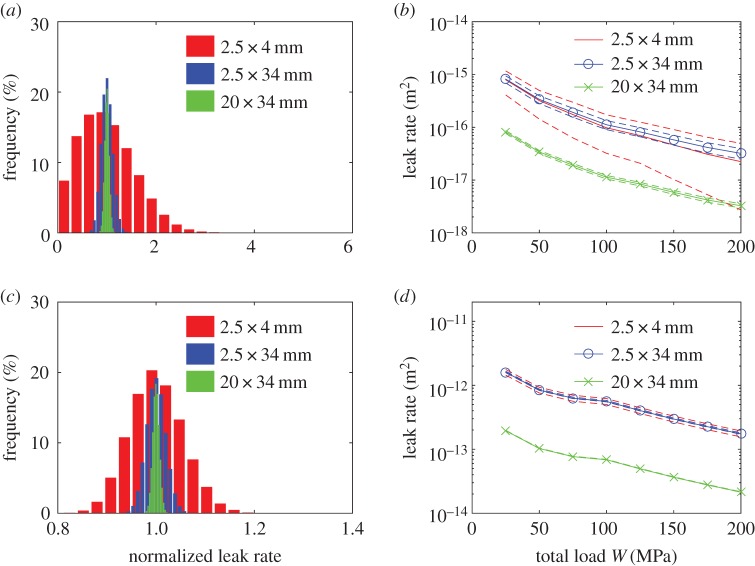
(*a*) Histogram of the leak rate obtained using Topography 1, normalized by its mean value, for three global domain size. The size is indicated as *x*_1_-direction × *x*_2_-direction. (*b*) Total flow per unit width as computed with the developed model using Topography 1 and with different global domain size. In dashed line, the corresponding 95% CI. (*c*,*d*) The same results as (*a*,*b*) obtained using Topography 2. (Online version in colour.)

The leak rate is computed for several permeability distribution realizations. When a sufficient number of realizations have been computed, the leak rate can be described statistically. The probability distribution for the leak rate for three global domain sizes can be seen in figure 11*a* for Topography 1 and in figure 11*c* for Topography 2. The results for Topography 1 have been computed using cells of width 0.09 mm, resulting on global domains of 12×48, 12×384 and 96×384 local cells in the *x*_1_- and *x*_2_-directions, respectively. Cells of width 0.18 mm have been used for Topography 2. When the leak rate is high, or when the global-scale domain is large compared with the local domain size, the distribution tends to be normal. However, as either of the leak rate or the global-scale domain become smaller, the variance of the leak rate distribution increases and it becomes skewed. If a normal distribution can be assumed for the leak rate, it can be characterized by using only the mean value and the standard deviation of that distribution. In the more general case where the distribution is not known, a 95% CI for the leak rate can be computed, as shown in figure 11*b* and *d*. Without knowing the distribution, this interval is obtained by performing a large number of computations until 95% of the computed leak rates fall consistently inside a fixed range. Some general trends can be observed with increase of global-scale domain. As it is increased in the *x*_2_-direction, a reduction of variability of the leak rate is observed. Both the reduction of variability and the tendency to become normal when increasing the size in the *x*_2_-direction are expected results as the effect of extreme values will be smaller and all realizations will be more similar due to the presence of more local cells. It is also notable that the leak rate per unit width is slightly increased in the case of Topography 1. This is because of the larger domain allow areas with high permeability to appear more often in the realizations. This effect is not observed in Topography 2 because its larger leakage allows for a better averaging even at the smallest domain. An increase of the global domain in the *x*_1_-direction has the opposite effect and mean leak rate is reduced. The reason for this is that the fluid must cover a longer distance and a narrower constriction is more likely to be encountered. One should expected the leak rate per unit width to converge to a certain value as the size of the domain in the *x*_1_ and *x*_2_-directions increases. It is noticeable, however, that convergence with size in the *x*_1_-direction is far from being reached even at realistic seal dimensions.

## 4. Concluding remarks

In pressure-driven flows, as the leak rate decreases, the fluid follows a pattern which becomes wide in the direction transverse to the pressure gradient. This obliges utilizing a large local domain in the common two-scale formulations. However, the required size of the local domain can become too large and approach the global domain, which makes it not possible to perform a scale separation of the flow. In order to avoid this problem, a two-scale model based on the HMM framework has been developed. This model does not assume periodic repetition of the topography and, therefore, can capture this wide flow pattern via local variation of permeability. This allows using much smaller local-scale domains. It has been shown that in this way a two-scales model which captures correctly the flow pattern can be developed.

Moreover, the presented two-scale formulation permits the construction of the local scale by considering the local permeabilities a random variable. This feature allows inclusion of the inherent uncertainty coming from the surface topography in the model and estimating the uncertainty on the leak rate due to this cause.

In the case study presented, it has been shown that smaller global-scale domains, as well as smaller leak rates, lead to more uncertainty on the predicted leak rate. It has also been shown that, even for relatively large global domains, the leak rate per unit width is dependent on the domain size. This has been explained as an effect of the random construction of the permeability distributions.

## Supplementary Material

Topography1

## Supplementary Material

Topography2

## Data accessibility

The datasets supporting this article have been uploaded as part of the electronic supplementary material.
